# Modifying Post-Operative Medical Care after EBV Implant May Reduce Pneumothorax Incidence

**DOI:** 10.1371/journal.pone.0128097

**Published:** 2015-05-26

**Authors:** Dominik Herzog, Alexander Poellinger, Felix Doellinger, Dirk Schuermann, Bettina Temmesfeld-Wollbrueck, Vera Froeling, Nils F. Schreiter, Konrad Neumann, Stefan Hippenstiel, Norbert Suttorp, Ralf-Harto Hubner

**Affiliations:** 1 Department of Internal Medicine/Infectious Diseases and Respiratory Medicine, Charité -Universitätsmedizin Berlin, Augustenburger Platz 1, 13353, Berlin, Germany; 2 Institute of Radiology, Charité - Universitätsmedizin Berlin, Augustenburger Platz 1, 13353, Berlin, Germany; 3 Department of Nuclear Medicine, Charité-Universitätsmedizin Berlin, Augustenburger Platz 1, 13553, Berlin, Germany; 4 Institute for Biometry and Clinical Epidemiology, Charité -Universitätsmedizin Berlin, Hindenburgdamm 30, 12203, Berlin, Germany; Weill Medical College of Cornell University, UNITED STATES

## Abstract

**Objective:**

Endoscopic lung volume reduction (ELVR) with valves has been shown to improve COPD patients with severe emphysema. However, a major complication is pneumothoraces, occurring typically soon after valve implantation, with severe consequences if not managed promptly. Based on the knowledge that strain activity is related to a higher risk of pneumothoraces, we asked whether modifying post-operative medical care with the inclusion of strict short-term limitation of strain activity is associated with a lower incidence of pneumothorax.

**Methods:**

Seventy-two (72) emphysematous patients without collateral ventilation were treated with bronchial valves and included in the study. Thirty-two (32) patients received standard post-implantation medical management (Standard Medical Care (SMC)), and 40 patients received a modified medical care that included an additional bed rest for 48 hours and cough suppression, as needed (Modified Medical Care (MMC)).

**Results:**

The baseline characteristics were similar for the two groups, except there were more males in the SMC cohort. Overall, ten pneumothoraces occurred up to four days after ELVR, eight pneumothoraces in the SMC, and only two in the MMC cohorts (p=0.02). Complicated pneumothoraces and pneumothoraces after upper lobe treatment were significantly lower in MMC (p=0.02). Major clinical outcomes showed no significant differences between the two cohorts.

**Conclusions:**

In conclusion, modifying post-operative medical care to include bed rest for 48 hours after ELVR and cough suppression, if needed, might reduce the incidence of pneumothoraces. Prospective randomized studies with larger numbers of well-matched patients are needed to confirm the data.

## Introduction

COPD, characterized by hyperinflation of the lung resulting in reduced gas exchange, is a major cause of morbidity and mortality worldwide. Endoscopic lung volume reduction (ELVR) with valves has been shown to improve lung function, quality of life and exercise capacity in severe COPD patients [1–9]. Pneumothoraces after ELVR are directly related to valve implantation [10] and result from the rapid shifts in volume between lung lobes. While the target lobe quickly loses volume following valve implantation, the adjacent ipsilateral untreated lobe expands to occupy the newly created space in the chest cavity. This volume shift can occur very quickly resulting in a parenchymal rupture of the untreated ipsilateral lobe [11]. The prevalence of pneumothorax after ELVR has recently increased due to stricter selection criteria of patients for valve therapy [12]. An absence of collateral ventilation [13], a heterogeneous emphysema [14] and a total occlusion of the target lobe have been shown to be predictive factors of better clinical outcome but also higher rates of pneumothoraces [15]. Although pneumothorax is a complication following ELVR, it seems not to have any negative impact on clinical outcomes [10].

There are also reports of correlations between strain activity, Valsalva maneuvers and the occurrence of pneumothoraces [16]. Nevertheless, data are still inconsistent and may not represent secondary pneumothoraces such as those following ELVR [17]. Interestingly, a recent paper identified biomechanical causes for spontaneous pneumothoraces in patients with a low thoracic index whose chests were transversely wider at the apex and flatter at the base. Mechanical stress was found to be 20 times higher in these patients during maximal breathing maneuvers such as coughing or strain activities [18]. Based on these reports and on our own observations of our patients that pneumothoraces were associated with physical activity and coughing after ELVR we asked: are patients less likely to develop pneumothoraces if their post-operative care is modified to limit physical activity with bed rest (BR) and cough suppression (CS) with codeine administered as needed, when added to standard post-implantation management?

## Methods

### Patients

Between October 2010 and March 2014, in total, 72 consecutive COPD patients with severe homogeneous or heterogeneous emphysema and negative collateral ventilation (CV) status assessed by the Chartis console (Pulmonx Inc., USA) were treated with endobronchial valves (Pulmonx Inc., USA) at the Charité Hospital in Berlin, Germany.

The eligibility criteria for ELVR treatment with valves included either male or female patients over 18 years of age diagnosed with severe lung emphysema GOLD stage III or IV and a nonsmoker status for at least six months as verified by several tests of carboxyyhemoglobin (HbCO) levels below 2.0%. The exclusion criteria were a forced expiratory volume in one second (FEV1) either below 15% of the predicted value (pred.) or more than 50% pred., a residual volume (RV) below 150% pred., a total lung capacity (TLC) below 100%, the presence of interlobar collateral ventilation by the Chartis assessment, a body mass index (BMI) more than 35 kg/m^2^, pregnancy or major comorbidities such as significant pulmonary hypertension or unstable cardiac conditions.

### Study design

**Fig 1:**
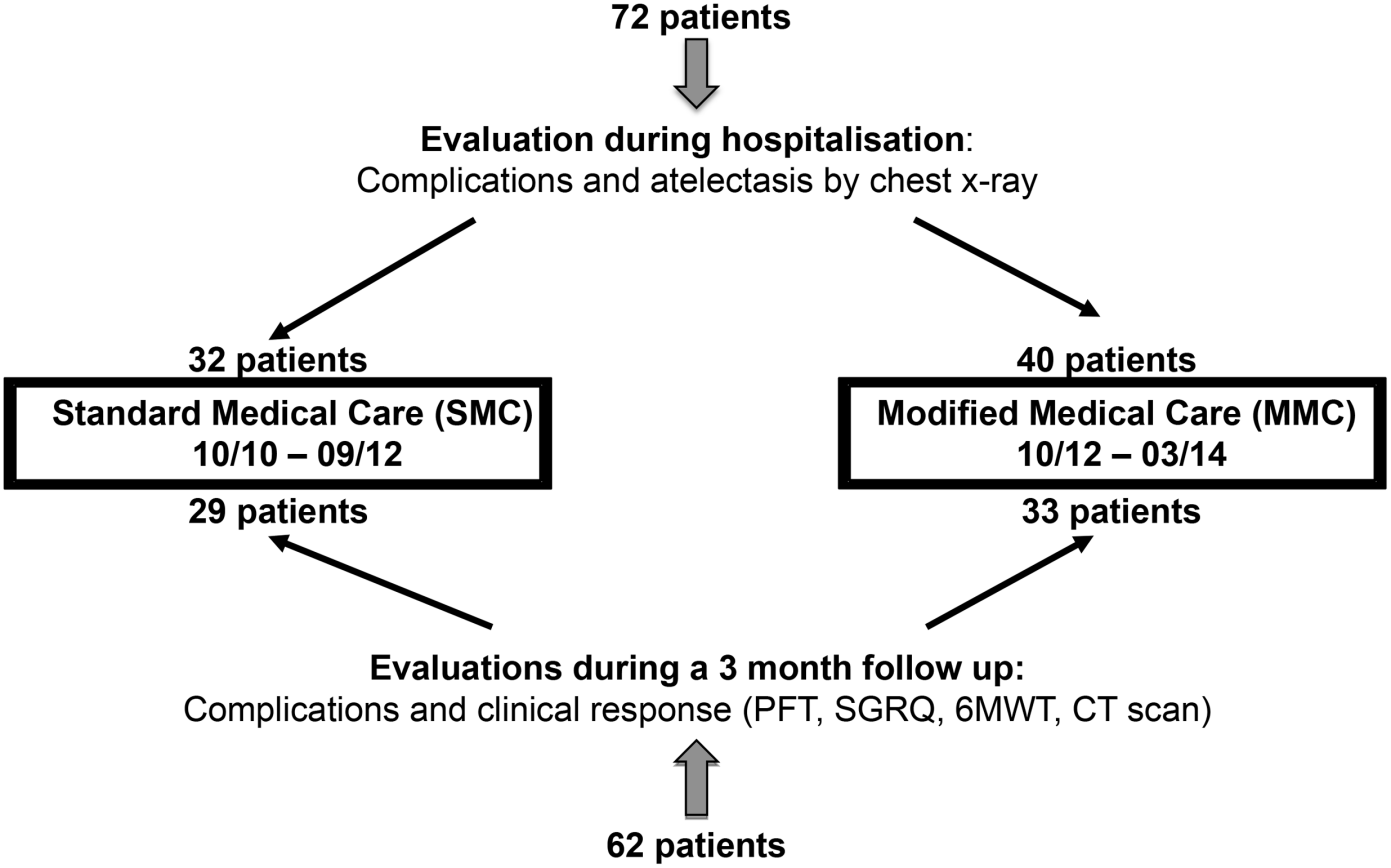
Study design. Seventy-two CV negative patients were treated with endobronchial valves at Charité Hospital between 2010 and 2014. In a retrospective study design, complications and development of an atelectasis were evaluated during hospitalization in all 72 patients comprising 32 patients with Standard Medical Care (SMC) (without restriction to bed rest) and 40 patients with Modified Medical Care (MMC) including strict 48 hour bed rest and cough suppression. Complication rate and clinical outcomes comprising pulmonary function, 6MWT and SGRQ during a three month follow up were assessed in 62 out of the 72 valve treated patients. CT scans at 3 months were available for 26 patients in the SMC and 29 patients in the MMC-cohort. CV: collateral ventilation. PFT: pulmonary function test. SGRQ: St. George respiratory questionnaire, TLVR: target lobe volume reduction, 6MWT: six minute walk test.

This study was a retrospective analysis. The study design was approved by the ethics committee of Charité University Hospital (E-No: EA1/280/14). Prior to analysis, patient records/information was anonymized and de-identified. Seventy two (72) consecutive patients were treated with endobronchial valves. Thirty-two (32) patients were treated with standard medical care (SMC) without restriction to bed rest and 40 patients followed a modified medical care (MMC) that included 48 hours strict bed rest and, if needed, 16 mg codeine up for cough to three times a day (TID). All SMC patients who were treated between October 2010 and September 2012 received the standard conventional postoperative management including post-interventional antibiotic treatment (sulbactam and ampicillin or moxifloxacin for five days) and thromboembolic prophylaxis, but with no restriction on BR and no cough suppression. The patients were asked to mobilize immediately after the valve implantation in order to avoid thromboembolic events. In the context that strain activity and coughing is reported to induce pneumothoraces and based on our clinical observation of a high rate of strain activity and coughing related to pneumothoraces under conventional postoperative management we decided to add BR for 48 hours to limit physical activity and, as needed, cough suppression with 16 mg Codeine up to TID to the standard conventional postoperative management. This new postoperative management procedure was applied to 40 patients treated between October 2012 and March 2014. There were only two patients in the SMC-cohort whose weakened conditions limited their taking of meal to their rooms. These two patients did not develop a pneumothorax. All complications and the development of an atelectasis during hospitalization were analyzed retrospectively from patient files and chest X-rays in all 72 patients (Fig 1A). Nine out of the 72 patients were treated twice after valve removal following pneumothorax (n = 1), severe infection (n = 2), leakage (n = 3) or valve expectoration (n = 3). These patients were included only once in the study and grouped exclusively with the last treatment into MMC or SMC. For example, one patient with a pneumothorax after the first treatment was treated again in September 2012 in the left upper lobe and developed another pneumothorax after the second treatment, both times without restriction to BR; thus, this patient was included only once in the SMC cohort. Another example is a patient who was initially treated in the left lower lobe in July 2012 without a pneumothorax but whose valves were removed because of migration and pneumonia two months later. This patient developed a pneumothorax after the second treatment in July 2013 on the second day after treatment of the left lower lobe despite BR; thus, this patient was included in the MMC cohort. Finally, no patient experiencing a pneumothorax was overlooked. Five patients received narcotic medication during hospitalization comprising three patients in the SMC-cohort who received Morphine 2% five drops per day, MST 10mg 1-0-1, Valoron N retard 150 12mg, 0-0-1 versus two patients in the MMC-cohort who received Tilidine/Naloxone 100mg 1-0-1 and Palladone 8mg 1-0-0/MST 10mg 1-0-1. The clinical outcomes and complications during a three month follow up were assessed in 62 out of 72 patients treated with valves. Ten patients did not receive a follow up examination because of previous valve removal (n = 4) or because patients refused further examinations after ELVR (n = 6).

### ELVR treatment

Before valve implantation, all patients had an outpatient visit for a physical examination, optimization of COPD medication, pulmonary function and diffusion tests (Weinmann, Germany), Six minute walk test (6MWT), St. George`s Respiratory Questionnaire (SGRQ) and blood gas analysis. The SGRQ ranges from 0 to 100, a higher score indicating a lesser quality of life. Patients also were sent to cardiologists to rule out significant pulmonary hypertension and unstable coronary heart disease. To evaluate lung destruction and emphysema distribution, patients received CT scans in a low dose technique without contrast agent prior to valve treatment. To ensure there were no differences in the number of homogeneous and heterogeneous emphysema patients between the two cohorts, which might have caused a bias in clinical outcomes and the probability of occurrence of pneumothorax, the emphysema score was analyzed with the MevisPulmo3D software (Fraunhofer, Bremen, Germany). Heterogeneous emphysema was defined as emphysema differing by more than 15% between the ipsilateral upper and lower lobes.

Collateral ventilation was assessed with the Chartis console in a bronchoscopic setting. Patients meeting the eligibility criteria were hospitalized one day prior to ELVR valve treatment and checked if they were in stable condition. In both cohorts, endobronchial valves (Zephyr, Pulmonx Inc.) were inserted unilaterally by the same bronchoscopist with a flexible bronchoscope into lobar, segmental or sub segmental bronchi according to the patient`s individual lung anatomy. The target lobe for treatment was the CV negative lobe with the highest emphysema distribution and/or lung destruction. For the bronchoscopic anaesthesia, midazolam and propofol were used. For ELVR all patients were admitted to the hospital and the hospital stay was similar in both groups (Table 1). In all patients, chest X-rays were taken before ELVR, one and three days after ELVR, and on the day of discharge to evaluate signs of lung volume reduction and to rule out a pneumothorax. All chest X-rays taken during hospitalization were retrospectively reassessed by two radiologists who were blinded to the study groups and results. Atelectatic signs in X-rays were defined as the detection of patchy opacities, mediastinal shift or elevation of the diaphragm. Patients were discharged in a clinically stable condition after at least four days following ELVR. A complicated pneumothorax was defined as a patient receiving a chest tube for treatment of a pneumothorax for more than five days. An exacerbation was defined as subjective dyspnea requiring a higher dosage of corticosteroids. Pneumonia was reported when patients had productive coughing with pus in their sputum, and who were administered antibiotics.

**Table 1:** Demographic characteristics of the study population^1^.

Parameter	SMC	MMC	p-value^2^
N	32	40	
Sex (male/female)	25/7	20/20	0.01
Age (yr)	68±1	69±1	0.9
BMI (kg/m^2^)^3^	25±1	24±1	0.2
Smoking history (pack-yr)	48±5	41±3	0.6
COPD stage			
GOLD III	7	10	0.8
GOLD IV	25	30	0.8
Emphysema distribution			
homogeneous	11	20	0.2
heterogeneous	9	14	0.1
Valves per patient	4±0	4±0	0.8
Treatment site^4^			
RUL	5	3	0.3
RLL	4	3	0.5
LUL	8	9	0.8
LLL	15	25	0.2
Pulmonary function test^5^			
FEV1	24±1	27±1	0.3
RV	217±10	223±7	0.4
DLCO	30±5	23±3	0.8
FVC	55±2	59±2	0.1
6MWT^6^	211.3±20	217.3±18	1.0
SGRQ^7^	63.2±3	67.4±2	0.5

^1^ Data are presented as mean ± standard error or number of patients

^2^ Group comparisons were made with the Mann-Whitney U test and the Chi Square test

^3^ Body mass index

^4^ RUL right upper lung, RLL right lower lung, LUL left upper lung, LLL left lower lung

^5^ Pulmonary function testing parameters are given as percent of predicted value; FEV1—forced expiratory volume in 1 sec, FVC—forced vital capacity, RV—residual volume, DLCO—diffusing capacity

^6^ Six minute walk test

^7^ St. George’s Respiratory Questionnaire

SMC: Standard Medical Care

MMC: Modified Medical Care

### Follow-up

At three months, patients received a physical examination, pulmonary function and diffusion tests, 6MWT, SGRQ and blood gas analysis. CT scans were performed in 26 patients of SMC group and 29 patients of the MMC cohort and analyzed with Pulmo3D to determinate the target lobe volume reduction (TLVR). A TLVR of more than 350ml was defined as a significant volume reduction. Complications after ELVR were assessed in a questionnaire that included exacerbations, pneumonia and other infections, hemoptysis, hospital stays, and thromboembolic events, among others.

### Statistics

The chi-square test and the Fisher Exact test for discrete variables were used for statistical analysis of categorical variables. The one-way analysis of variance (ANOVA) was used to compare atelectasis scores between the two groups. The Man-Whitney-U test was used for ordinal variables. A p value <0.05 was considered statistically significant. A logistic regression analysis with the factors study group and gender was performed in order to adjust for a possible influence of gender on the occurrence of pneumothorax. The data are displayed as mean +/- standard error. SPSS (IBM, version 20) was used for all statistical analyses.

## Results

### Patients

Seventy-two patients were treated with endobronchial valves, comprising 32 patients without restriction to bed rest (SMC cohort)) and 40 patients with 48 hours strict bed rest, and if needed for cough, 16 mg codeine up to TID (MMC cohort). The baseline characteristics are listed in Table 1. Both cohorts were similar with regard to age (p = 0.9), BMI (p = 0.2), smoking history (p = 0.6), disease stage (p = 0.8), emphysema distribution (p = 0.2), number of inserted valves (p = 0.8), treatment site (p = 0.2), pulmonary function and diffusion tests, including FEV1 (p = 0.3), FVC (p = 0.1), RV (p = 0.4), 6MWT (p = 1.0) and SGRQ (p = 0.5). However, there was a significant difference in the sex ratio: 25 patients (78%) of SMC but only 20 patients (50%) of MMC were males (p = 0.01).

### Complications during Hospitalization

**Fig 2:**
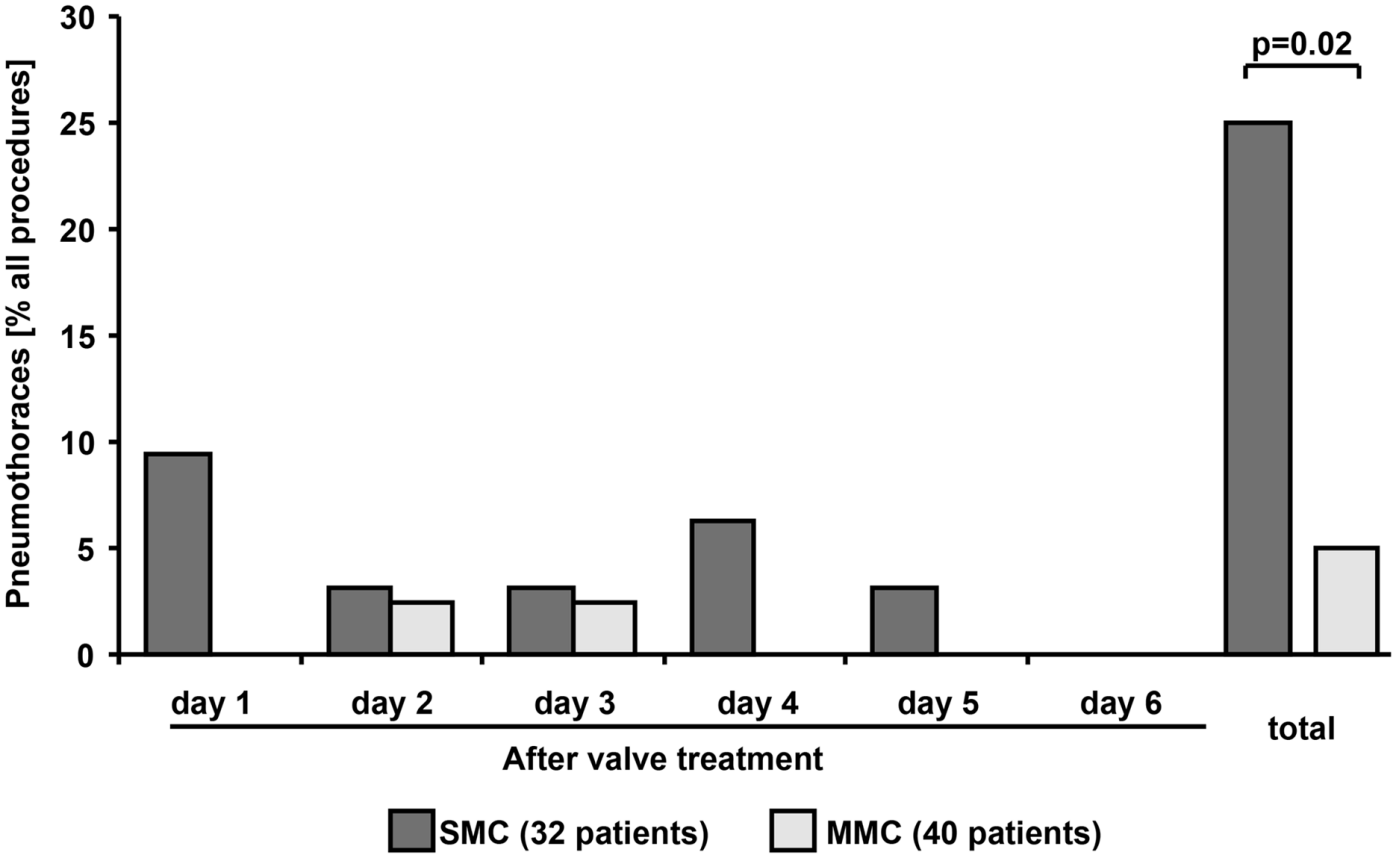
Incidence of pneumothorax after ELVR. Pneumothoraces occurred less often in the Modified Medical Care (MMC) than in the Standard Medical Care (SMC) cohorts.

To evaluate complications with a focus on the pneumothorax rate, patient files and chest X-rays taken during hospitalization were assessed retrospectively. Hospitalization time was equally long in both cohorts (p = 0.1, Table 2). There were no deaths following ELVR with valves. In total, 10 pneumothoraces occurred over both cohorts during hospitalization, all on the ipsilateral side of the treated lobes and within four days after the procedure. Overall, compared to MMC, SMC demonstrated significantly more pneumothorax occurrence (25% (n = 8) for SMC vs 5% (n = 2) for MMC, p = 0.02, Fig 2), particularly with respect to upper lobe treatments (p = 0.008, Table 2). SMC more often had complicated pneumothoraces characterized by drainage therapy lasting longer than five days (p = 0.02) and more frequently developed broncho pleural fistula (p = 0.04), compared to MMC. In SMC, three patients were transferred with respiratory failure to the ICU because of pneumothoraces and one MMC patient because of pneumonia and pleural effusion (p = 0.2). Valves were removed in four patients during hospitalization: three SMC patients because of pneumothorax and one MMC patient because of pleural effusion and pneumonia, defined as productive coughing with pus in the sputum and administered antibiotic therapy. In both groups there were no significant differences in other complications including exacerbations defined as subjective dyspnea requiring a higher dosage of corticosteroids (p = 0.8), pneumonia (p = 0.4), pleural effusion (p = 0.8), hemoptysis (p = 0.3) and thromboembolic events (p = n.a). Although there is a significant difference in the sex ratio between the two cohorts (p = 0.01) and pneumothoraces occurred significantly more frequent in the SMC-cohort (p = 0.02) there was no significant association between the sex ratio and the occurrence of pneumothoraces in our study cohort (p = 0.7). In order to adjust for a possible influence of gender on the occurrence of pneumothorax we performed a logistic regression analysis with the factors study group and gender. Again, gender had no significant effect on the occurrence of pneumothorax following ELVR (p = 0.9) whereas the SMC and MMC factor remained significant in the regression analysis (p = 0.02).

**Table 2:** Complications during hospitalization^1^.

	SMC	MMC	p-value^2^
Patients	32	40	
Hospital stay (days)	7±1	7±1	0.1
Death	0	0	n.a
Pneumothoraces^3^	8	2	0.02
After UL treatment	6	0	0.008
After LL treatment	2	2	0.4
Complicated pneumothoraces^4^	4	0	0.02
Bronchopleural fistula	3	0	0.048
Transfer to ICU^5^	3	1	0.2
Chest tube	7	1	0.01
Duration chest tube (days)	7±2	3	n.a
Pleural effusion	2	3	0.8
Exacerbations	8	8	0.8
Pneumonia	1	3	0.4
Thromboembolic events	0	0	n.a
Hemoptysis	1	0	0.3
Valves removal	3	1	0.2

^1^ Data are presented as mean ± standard error or number of patients

^2^ Group comparisons were made with the Mann-Whitney U test or the Fisher`s exact test

^3^ UL Upper lobe LL lower lobe

^4^ Defined as drainage >5d

^5^ ICU Intensive care unit

SMC: Standard Medical Care

MMC: Modified Medical Care

### Complications occurring between discharge after ELVR and three-months follow-up

To examine whether MMC was associated with a higher complication rate after hospital discharge, complications within three months after valve implantation were assessed in 62 out of the 72 patients There was one death of unknown cause in a SMC patient two months after ELVR. It is noteworthy that this patient had not developed an atelectasis after ELVR by the day of discharge. One patient in MMC who had a pneumothorax during hospitalization developed a second pneumothorax on the ipsilateral side of the treated lobe two months after ELVR. It was treated again with a chest tube over seven days and a full re-expansion of the lung was achieved. In summary, there were no significant differences between groups with respect to complications after hospital discharge (Table 3).

**Table 3:** Complications between discharge after ELVR and three-months follow-up^1^.

	SMC	MMC	p-value^2^
Patients	29	33	
Follow-up (months)	3.1±0.3	3.3±0.2	0.4
Death	1	0	0.3
Pneumothorax	0	1	0.3
Hospitalization	4	7	0.6
Duration hospitalization (day)	4±2	6±2	0.5
Exacerbation	16	12	0.1
Pneumonia	12	7	0.1
Thromboembolic events	0	0	n.a
Hemoptysis	3	1	0.1
Valves removal	7	8	0.9

^1^ Data are presented as mean ± standard error or number of patients

^2^ Group comparisons were made with the Mann-Whitney U test or the Fisher’s exact test

SMC: Standard Medical Care

MMC: Modified Medical Care

### Comparison of clinical outcomes after ELVR

**Fig 3:**
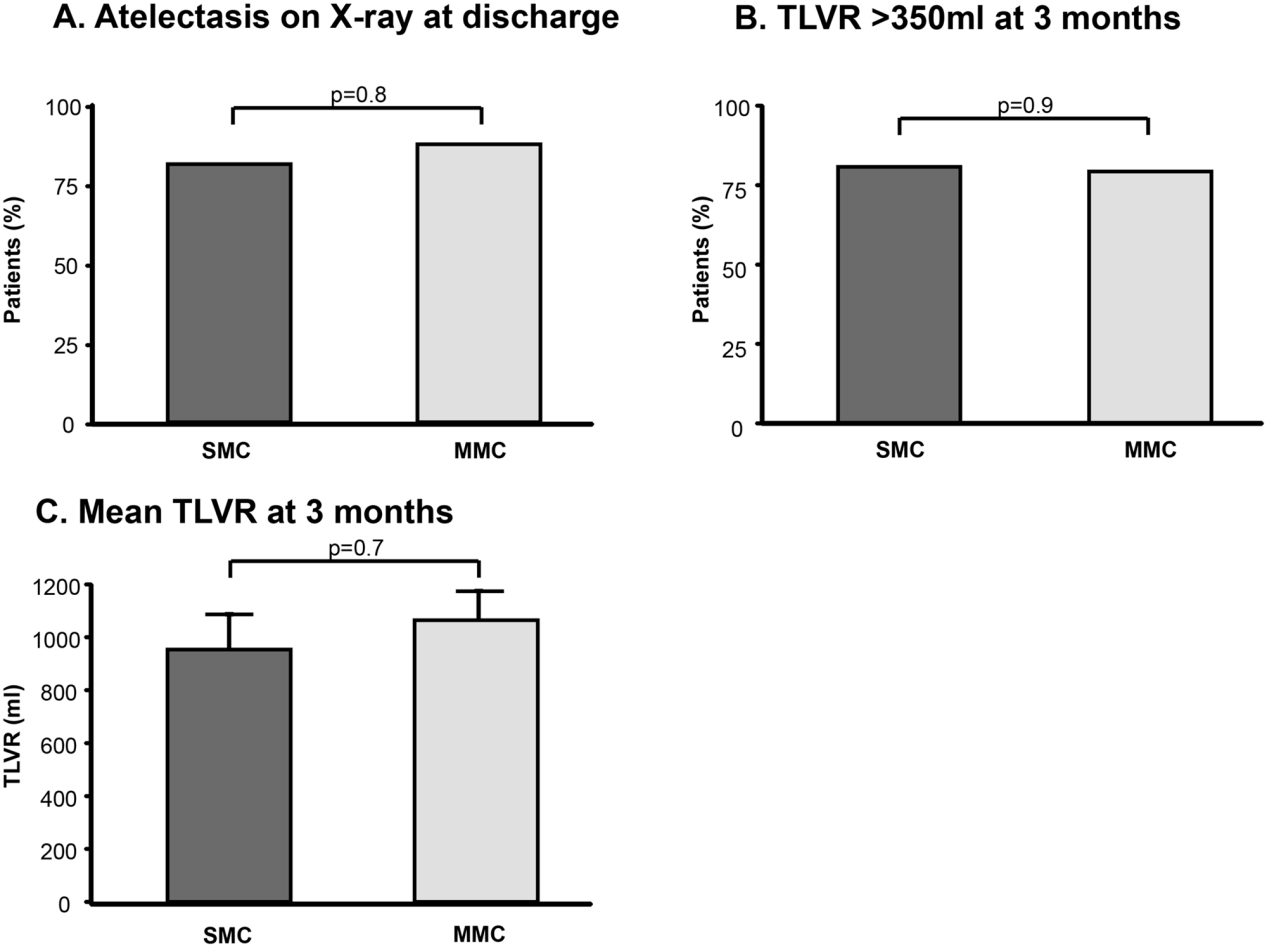
Radiological changes after ELVR. **A.** Atelectasis at chest X-ray on the day of discharge from hospital. **B.** Patients with a significant TLVR of more than 350ml at 3 months. **C.** Mean TLVR at 3 months. TLVR: target lobe volume reduction. SMC: Standard Medical Care. MMC: Modified Medical Care.

**Fig 4:**
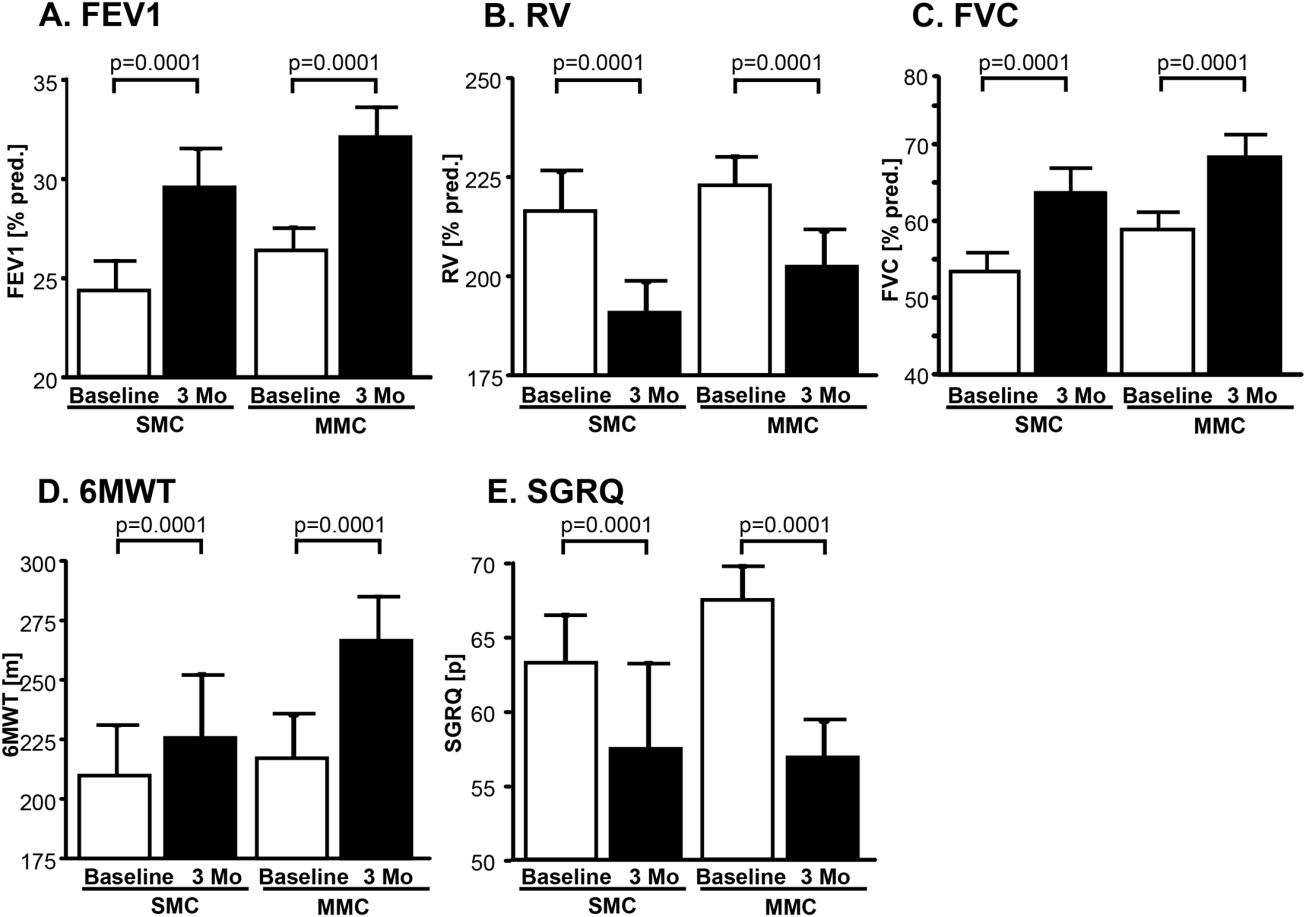
Clinical outcomes after three months compared to baseline. **A.** Forced expiratory volume in 1 sec (FEV1). **B.** Residual volume (RV). **C.** Forced vital capacity (FVC), **D.** Six Minute walk test (6MWT). **E.** St. George`s Respiratory Questionnaire (SGRQ). SMC: Standard Medical Care. MMC: Modified Medical Care.

At hospital discharge, 81% of SMC patients and 87% of MMC patients developed radiological signs for lung volume reduction (p = 0.8, Fig 3a). At three months follow-up, 81% of SMC patients and 79% of MMC patients had a significant target lobe volume reduction (TLVR) of more than 350ml as assessed by CT scans (p = 0.9, Fig 3b) with a mean volume reduction of 953±134ml in the SMC-cohort and 1065±110ml in the MMC-cohort (p = 0.7, Fig 3c). SMC and MMC also showed a similar increase of FEV1 (p = 0.7, Fig 4a, Table 4), FVC (p = 0.3; Fig 4c, Table 4) and likewise a similar decrease of RV (p = 0.3, Fig 4b, Table 4). Similar improvements in 6MWT (p = 0.5, Fig 4c, table 4) and SGRQ (p = 0.1, Fig 4d, table 4) were recorded.

**Table 4:** Comparison of clinical outcomes at 3 months after ELVR^1^ vs. baseline.

	SMC	MMC	p-value^2^
Patients	29	33	
FEV1 (% from baseline)^3^	+21.4±1	+22.7±4	0.7
RV (% from baseline)^4^	-10.0±4	-6.6±4	0.3
FVC (% from baseline)^5^	+22.1±5.1	+18.7±5.7	0.4
6MWT (m from baseline)^6^	+23.2±14	+38.5±12	0.5
SGRQ (points from baseline)^7^	-4.7±4	-12.0±2	0.1

^1^ Data are presented as mean ± standard error or number of patients

^2^ Group comparisons were made with the Mann-Whitney U test or the Fisher’s exact test

^3^ FEV1—forced expiratory volume in 1 sec

^4^ RV—residual volume

^5^ FVC—forced vital capacity

^6^ Six minute walk test

^7^ St. George’s Respiratory Questionnaire

SMC: Standard Medical Care

MMC: Modified Medical Care

## Discussion

The major complication directly related to valve implantation is pneumothoraces that could have serious sequelae if not treated promptly [11]. Out of 72 patients, 10 pneumothoraces occurred within the first four days after valve implantation, all on the ipsilateral side of treatment. We recorded a strikingly lower rate of pneumothoraces in MMC in comparison to SMC, and in particular after upper lobe treatment, there were less complicated pneumothoraces and fewer bronchopleural fistulae. Other complications during hospitalization, clinical and radiological outcomes during the three-month follow-up period remained similar in both groups.

It is suggested here that pneumothoraces might be related to strain activity; however, the evidence available in the literature is inconclusive. Cran et al. [19] analyzed retrospectively 994 pneumothorax cases in the Royal Air Force. In 358 patients strain activity was recorded at the time of onset. In the same study more pneumothoraces were seen during the winter, and interestingly, a correlation with coughing was recorded in 120 patients. Another publication describes pneumothoraces during increased Valsalva maneuvers, as seen in playing the trumpet or other musical instruments or blowing up balloons [16]. Interestingly, a recent paper evaluated the biomechanical forces during breathing and coughing and calculated a 20 times increased mechanical stress for spontaneous pneumothoraces during expiration and coughing in patients whose chests were transversely wider at the apex and flatter at the basis [18]. In contrast, Bense et al. [17] found in 219 pneumothorax patients, retrospectively, that only 2% had moderate exertion at the time when pneumothorax occurred and no patients were exerting themselves heavily when the symptoms began.

The physiological cause of pneumothoraces directly related to valve implantation are hypothesized to result from the rapid shifts in volume between lung lobes [11]. While the target lobe quickly loses volume after valve implantation, the adjacent ipsilateral, untreated lobe expands to occupy the newly created space in the chest cavity. This volume shift can occur very quickly, resulting in a parenchymal rupture of the untreated ipsilateral lobe. We have seen in our patients some pneumothoraces associated with strain activity and coughing that we think may be among many other important contributing risk factors for pneumothoraces after ELVR.

In our study, we observed a high pneumothorax rate of 25% in patients with severe emphysema, no collateral ventilation, no restriction to bed rest and no specific administration of cough suppression drug following ELVR. This pneumothorax rate was higher than those reported in VENT study (4.2% [3]) and Euro-VENT study (5% [2]). However, pneumothorax criteria were different between these studies and ours: the VENT study only counted severe pneumothoraces that lasted longer than 5 days while we counted all pneumothoraces irrespective of severity. Only about one third of VENT and Euro-VENT patients had complete fissures, which explains that FEV1 increased an average of only by 4.3% (VENT) and 7.0% (Euro-VENT) and the mean TLVR was only 378.4ml in the VENT patients (no data for Euro-VENT patients). In the SMC-cohort, however, 81% of all patients had complete fissures and thus developed atelectasis during hospitalization: the FEV1 increased by +21.4±1% from baseline and the mean TLVR was 953±134ml. The improved outcomes based on better selection criteria for the patients also imply a higher risk for pneumothoraces. Many other groups recently reported higher pneumothorax rates with respect to improved selection criteria: 23% Gompelmann et al.[20], 20% Lesser et al [21]., 23% Bosc et al [22] and 17% Gieserich et al [23].

Despite the striking differences between our two groups with regard to the prevention of pneumothoraces, these data need careful interpretation as many caveats may be present. It is possible that the reduction of pneumothoraces is the result of other factors that were not considered in this study. Another important study limitation is its retrospective analyses design, such that selection bias cannot be excluded and that the only data that were examined were those recorded in the patients’ files. Another limitation is that baseline characteristics were different with regard to sex ratio. For unknown reasons, significantly more males were treated in the MMC group. Nevertheless, despite the higher risk of spontaneous pneumothorax for males [24–27], our analysis shows no evidence that males are more susceptible to pneumothoraces after ELVR than females.

Although data were available for a large number of treated patients, the total number of pneumothoraces is in fact very small. It is important to remember that in this context small changes in events may have large effects on the outcome of the study. Another problem is that the confinement of patients to bed rest for 48 hours to prevent physical activity is a change of paradigm from clinical practice that favors early mobilization. This confinement might be associated with a greater rate of side effects, such as pneumonia or thromboembolic events. Notwithstanding, there were no significant differences in any of the recorded complications of exacerbation, rehospitalization, thromboembolic events and hemoptysis between the two groups during hospitalization and follow-up. Although not statistically significant, we have seen one late onset of pneumothorax in the MMC cohort occurring two months after ELVR and none in the SMC cohort. Studies with a higher number of patients are needed to investigate if bed rest avoids or in some cases delays the occurrence of pneumothoraces which may be more dangerous as patients may develop these episodes in an outpatient setting. There was no further evaluation of the impact of the duration of bed rest. We observed that most pneumothorax events occurred within the first 48 hours after ELVR, which was our rationale for keeping patients in bed for this duration. However, it might be possible that shorter or longer bed rest times might be equally or more effective in the reduction of pneumothoraces. We do not know whether cough suppression with codeine or bed rest was more relevant in preventing pneumothoraces. Due to the retrospective study design we do not know if the patients in the MMC-cohort really coughed less with the codeine-treatment than the patients in the SMC-cohort and thus the degree and efficiency of cough suppression remains unclear. For the treatment and analyses of future patients with valves we have implemented a cough score. As the two cohorts were not treated at the same time period, improvements in technique of the bronchoscopist may have influenced the clinical outcomes and complication rates. Nevertheless, we have seen similar clinical and radiological outcomes in both cohorts, thus minimizing the possibility of a training effect.

In summary, modifying post-operative care to include a 48-hour bed rest and cough suppression with codeine, if needed, was found to reduce the pneumothorax rate after ELVR with valves, while other complications and clinical outcomes remained the same in both cohorts. These observations suggest that strain activity and other Valsalva maneuvers may be risk factors for pneumothoraces following ELVR. However, a prospective, randomized study with a larger number of patients and similar baseline characteristics are needed to confirm the outcomes of this study.
